# Interpersonal heart rate synchrony and prosocial behaviors in musical contexts

**DOI:** 10.3389/fpsyg.2026.1836447

**Published:** 2026-08-04

**Authors:** João Marcos Parolo Carmona, Antonio Pereira, Rael Bertarelli Gimenes Toffolo, Felipe de Oliveira Matos

**Affiliations:** 1 Graduate Program in Physiological Sciences, State University of Maringá (UEM), Maringá, Brazil; 2 Signal Processing Laboratory, Institute of Technology, Federal University of Pará (UFPA), Belém, Brazil; 3 Center for Music and Music Therapy, State University of Paraná, Maringá, Brazil; 4 Department of Physiological Sciences, State University of Maringá (UEM), Maringá, Brazil

**Keywords:** auditory stimulation, cardiac activity, cognitive processing, music, physiological signals, social connection

## Abstract

The interpersonal synchronization of physiological signals has been a major target of cognitive science studies, mainly because it is associated with pro-social behaviors. In this context, naturalistic stimuli are often used to evaluate this effect, using neuroimaging techniques. However, it is also possible to observe synchronizing effects at the cardiac level, which may also reflect cognitive processing. The main goal of this mini-review is to achieve a better understanding of interpersonal heart rate synchronization and pro-social behaviors in musical contexts. We conclude that music has the ability to strengthen the sense of connection and collaboration between people, being a facilitator of interpersonal synchronization at various physiological levels, especially in heart rate.

## Introduction

Music is a universal art that transcends time, cultural and linguistic frontiers. Beyond its entertainment value and capability to evoke emotions, music has garnered increasing attention in the scientific community for its potential impact on human behavior. Besides providing aesthetic pleasure, enjoyment, and interaction between individuals, music plays a crucial role in emotional expression, establishment of social bonds, and the strengthening of interpersonal connections (Savage et al., 2021). A particularly compelling area of inquiry involves the extent to which music can stimulate or modulate prosocial behaviors—actions that contribute to the welfare of others or society as a whole.

Through group activities such as dancing or singing, shared musical experiences have the potential to bring people together (Cirelli et al., 2014). This observation is supported in a study with children, which demonstrated that helping and sharing behaviors were facilitated by music making (Beck and Rieser, 2022), emphasizing the role of music in promoting prosocial actions even from the early stages of human development (Cirelli et al., 2017).

Recent evidence indicates that engaging in prosocial behaviors boosts personal happiness, reinforces social bonds, and enhances overall well-being (Lanser and Eisenberger, 2023). Beyond these immediate gains, prosocial actions are associated with higher subjective well-being and elevated social status, advantages that may translate into an evolutionary edge by fostering group cohesion and survival (Wang, 2022; Labroo et al., 2023).

A recent literature review by Müller et al. (2021) proposed a potential physiological explanation for prosocial behaviors, emphasizing the synchronization of multiple variables, such as brain activity and heart rate, both within individuals and between them. This fascinating phenomenon suggests the existence of a neural mechanism that not only supports interpersonal synchronization but also coordinates collective actions aimed at achieving shared goals. However, understanding physiological interpersonal synchronization at the physiological level requires a thorough analysis of the interaction among several central nervous system’s circuits associated with perceptual, cognitive, emotional, and motor capabilities (Kleshchova and Weierich, 2021). Furthermore, special attention should also be given to the autonomic nervous system (ANS), which plays a crucial role by coordinating visceral responses and integrating feedback on internal physiological states through vagal afferents (Porges, 2007).

In this mini-review, we revisit some notable findings on music and the synchronization of physiological variables. Particular emphasis was placed on the role of the ANS as a key player underlying the complex relationship between music and prosocial behavior, providing valuable insights for fields such as social psychology, neuroscience, psychophysiology, and music therapy.

### Music in the physiology of behavior

Humans have evolved and thrived in diverse environments largely due to their remarkable capacity for widespread social cooperation, which extends beyond immediate social circles (Boyd and Richerson, 2009). This distinguishes humans from other primates, who generally cooperate only within their own group (Sterelny, 2012). In this framework of extended cooperation, music has played a crucial role in fostering intergroup connections, acting as a powerful medium for expressing shared ideas and emotions across different communities (Wilson, 2013). Studies support that music likely emerged during human evolution as a mechanism for establishing and reinforcing social bonds among interacting group members (Kogan, 1997; Dunbar, 2012). However, the precise constraints and adaptations underlying these connections remain unresolved (Tarr et al., 2014), making research in this area particularly intriguing.

### Interpersonal (motor and physiological) synchronization

Physiological synchronization refers to the coordination and temporal alignment of physiological processes between individuals or within groups. This phenomenon can be observed in various variables, including heart rate (HR), respiratory cycles, skin conductance, neural activity, and endocrine responses such as cortisol and oxytocin levels (Palumbo et al., 2017; Goldstein et al., 2017; Tomashin et al., 2022; Jackson et al., 2018). These synchronizations reflect deeper mechanisms through which individuals align their physiological states during social interactions, fostering cohesion and cooperation. Such synchronization can occur under various conditions, including the production of individual rhythms or oscillations, where physical, chemical, or coupling interference may further influence or enhance this alignment (Strogatz, 2003).

Synchronization in humans is a prevalent characteristic, occurring even before birth, as demonstrated by the high degree of cardiac and respiratory synchronization between the mother and the fetus (Van Leeuwen et al., 2009). Another example later in life occurs during conversations, when the brainwaves of listeners tend to synchronize with those of the speaker (Kawasaki et al., 2013). These interpersonal physiological connections can manifest spontaneously, without the need for synchronization instructions (Demos et al., 2012), intimate relationships, or a shared goal between individuals, and they may occur independently of one another (Codrons et al., 2014). Additionally, simultaneous exposure to the stimulus is not a requirement for physiological systems to synchronize (Steiger et al., 2019; Madsen et al., 2021; Pérez et al., 2021).

One mechanism that has been proposed to underlie the synchronization of physiological signals is the mirror-neuron system (MNS), a network of premotor and posterior-parietal neurons that fires both when an individual executes an action and when they perceive the same action performed by others (Gallese et al., 1996; Rizzolatti et al., 1996; Rizzolatti and Craighero, 2004). Neurons within the MNS respond not only to visual but also to auditory representations of action (Keysers et al., 2003), and their activity is modulated by the actor’s emotional expression, the transparency of their intentions, and the observer’s aesthetic appraisal (Tanaka, 2021). The system shows stronger activation when the movement is performed by a biological agent (Calvo-Merino et al., 2005), particularly when the observer has prior sensorimotor experience with that action (Cannon et al., 2014). Based on this evidence, it is likely that the MNS contributes to the complex and intriguing human ability to connect not only behaviorally and emotionally but also biologically with other individuals of the same species.

**Figure 1:**
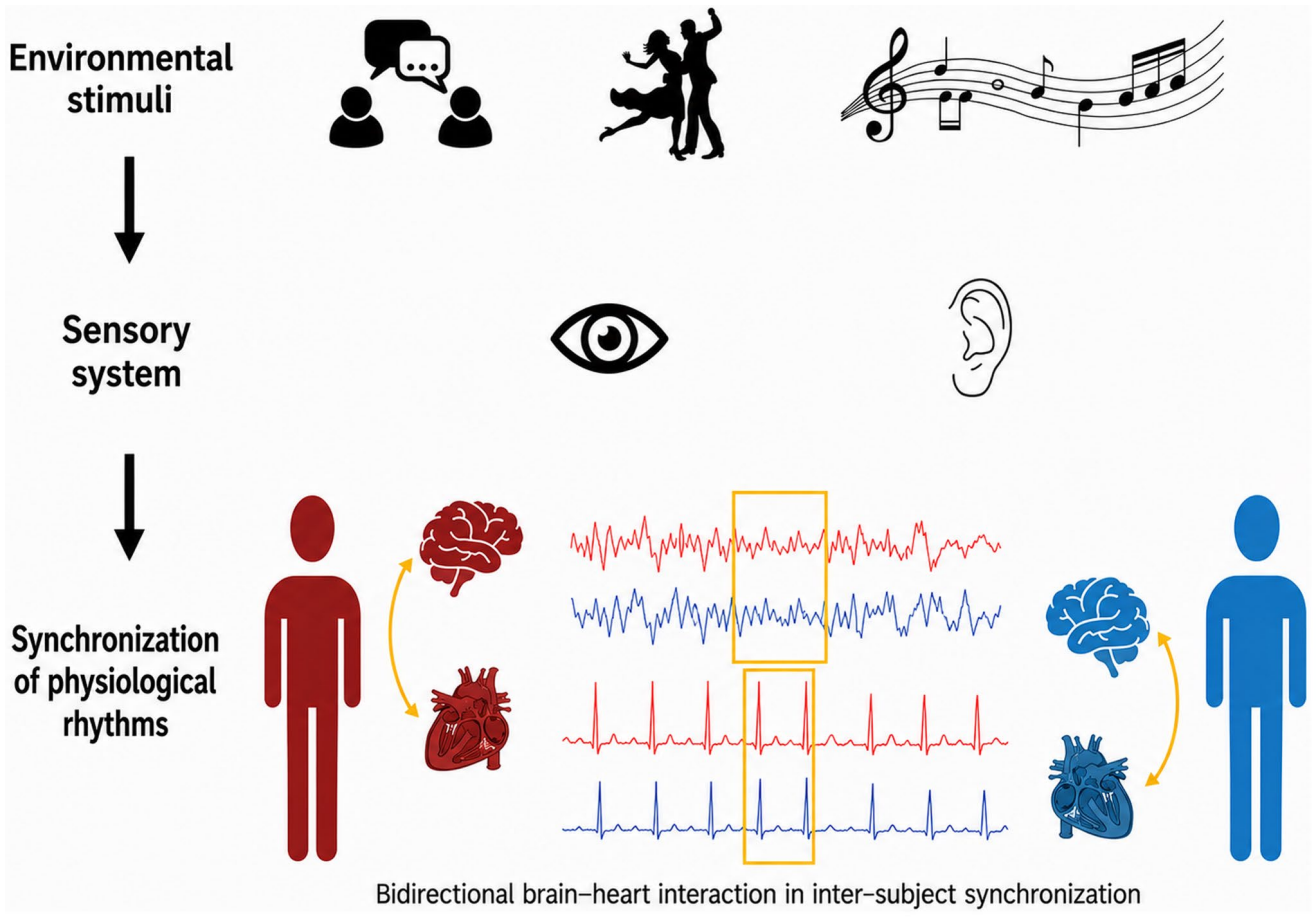
Conceptual diagram of the interpersonal and intrapersonal synchronization process. The image illustrates how environmental stimuli influence and synchronize physiological processes across individuals. Initially, external elements such as social interaction, movement (e.g., dance), and music act as environmental stimulus. These stimuli are perceived by the sensory system, particularly through vision and audition. Subsequently, this information is processed by the organism through internal mechanisms, including the bidirectional interaction between the brain and the heart. This reciprocal evaluation enables both physiological adjustments and the shaping of perceptual responses to stimuli. Such processing influences synchronization and elucidates shared experience through the alignment of biological responses among individuals, thereby promoting connection and coordination.

Figure 1 presents a conceptual model of interpersonal synchrony (IS), illustrating how shared environmental stimuli, such as social interactions, dance, and music, are processed through individuals’ sensory systems and contribute to the temporal alignment of physiological processes. The schematic highlights the role of sensory modalities, particularly vision and audition, in mediating this process, as well as the bidirectional interaction between the brain and the heart, which supports the synchronization of physiological rhythms across individuals. Overall, the figure summarizes the mechanisms through which shared experiences may promote interpersonal coupling at multiple biological levels.

### Music and interpersonal synchronization

The adaptive significance of music has been a longstanding focus of evolutionary and ethnomusicological inquiry (Dissanayake, 2008). By providing a temporally structured and affect-laden soundscape, music offers a fertile context for social interaction (Stupacher et al., 2022) and is thought to foster group cohesion and prosocial behavior (Huron, 2001; McDermott, 2008; Savage et al., 2021). Empirical work on interpersonal motor coordination further shows that synchronizing movements to a shared musical pulse strengthens social, emotional, and cooperative bonds among participants (Keller et al., 2014; Stupacher et al., 2017; Engel et al., 2022).

A growing literature demonstrates that listening to music also aligns autonomic responses across individuals, producing highly similar temporal profiles of cardiovascular, respiratory, and electrodermal activity (Bernardi et al., 2006; Bernardi et al., 2009; Bernardi et al., 2017; Lange et al., 2022; Gibbs et al., 2023). At the central level, music entrains activity from early sensory cortices to higher-order associative areas: electroencephalography (EEG) studies reveal stimulus-locked phase alignment of cortical oscillations (Abrams et al., 2013; Madsen et al., 2021), while rhythm perception engages motor circuitry even in the absence of overt movement (Engel et al., 2022). Gamma-band activity in right motor and parietal regions and theta activity in limbic structures track the affective impact of rhythm and melody (Lu et al., 2021; Chabin et al., 2022).

The properties of the musical stimulus shape both behavioral and physiological synchrony. Exposure to unfamiliar styles helps maintain listeners’ engagement (Madsen et al., 2019), whereas rhythmically simpler patterns are optimal for fostering motor entrainment within a group (Codrons et al., 2014). In contrast, repeated exposure generally reduces interpersonal synchronization, presumably because attention and affective arousal habituate over time (Madsen et al., 2019). Notably, music that listeners subjectively prefer evokes consistently stronger emotional and autonomic responses than non-preferred selections (Blood and Zatorre, 2001; Brattico and Jacobsen, 2009).

In musical practice, higher IS of physiological variables predicts better collective performance in rhythmic drumming (Kokal et al., 2011), choral singing (Müller and Lindenberger, 2011), and whole-body coordination tasks (Demos et al., 2012). Expertise further shapes synchronization: trained musicians align more precisely to external rhythms, adapt more rapidly to tempo changes, and show structural as well as functional enhancements throughout the auditory pathway (Repp, 2010; Kraus and Chandrasekaran, 2010; Karpati et al., 2016; Scheurich et al., 2020). Anticipatory timing skills also differentiate skilled from unskilled performers in joint sensorimotor tasks (Pecenka and Keller, 2011), while intrinsic (endogenous) rhythms contribute to IS in both groups (Zamm et al., 2016; Tranchant et al., 2022).

At the neural level, interpersonal coordination is supported by densely interconnected sensory-motor networks that integrate self-generated and observed actions. Although this integration occurs within each individual brain, it can give rise to inter-brain coupling—compatible modulations of sensorimotor activity across people—that underpins both spontaneous and deliberate forms of synchrony (Ménoret et al., 2014; Keller et al., 2014).

Despite substantial progress, physiological, biochemical, and genetic substrates linking music to social and cognitive outcomes remain incompletely understood. We anticipate that forthcoming advances in multimodal neuroimaging, large-scale data analytics, and molecular techniques will illuminate these mechanisms, clarifying how musical engagement shapes — and is shaped by — human biology and behavior.

### Music and the heart

Musical stimuli reliably evoke affective states through coordinated activity in subcortical reward and salience structures—including the ventral striatum and amygdala—and hierarchically organized auditory cortices (Liégeois-Chauvel et al., 2014; Salimpoor et al., 2015). A substantial portion of the information that music communicates is therefore mediated by the emotions it provokes (Juslin and Sloboda, 2010). Because emotions bias attention, modulate conscious experience, and orchestrate action (Tooby and Cosmides, 2008), the visceral component of these states—produced by sympathetic-nervous-system (SNS) outflow — is reflected in heart rate variability, for instance (McCraty and Shaffer, 2015).

Auditory stimulation elicits systematic autonomic adjustments, most prominently in the cardiovascular domain (Hilz et al., 2014), supporting the view that music functions as a regulator of emotion and behavior. Embodied-cognition theories posit that the motor apparatus and the visceral autonomic nervous system constitute core scaffolds for cognitive development, such that concepts and affects are grounded in coupled sensorimotor and autonomic networks (Leman and Maes, 2014). Mechanistically, this grounding is often framed in terms of bidirectional mapping between perception and action: auditory information is transformed into motor representations via mirror-mapping processes (Rizzolatti et al., 2001).

**Figure 2:**
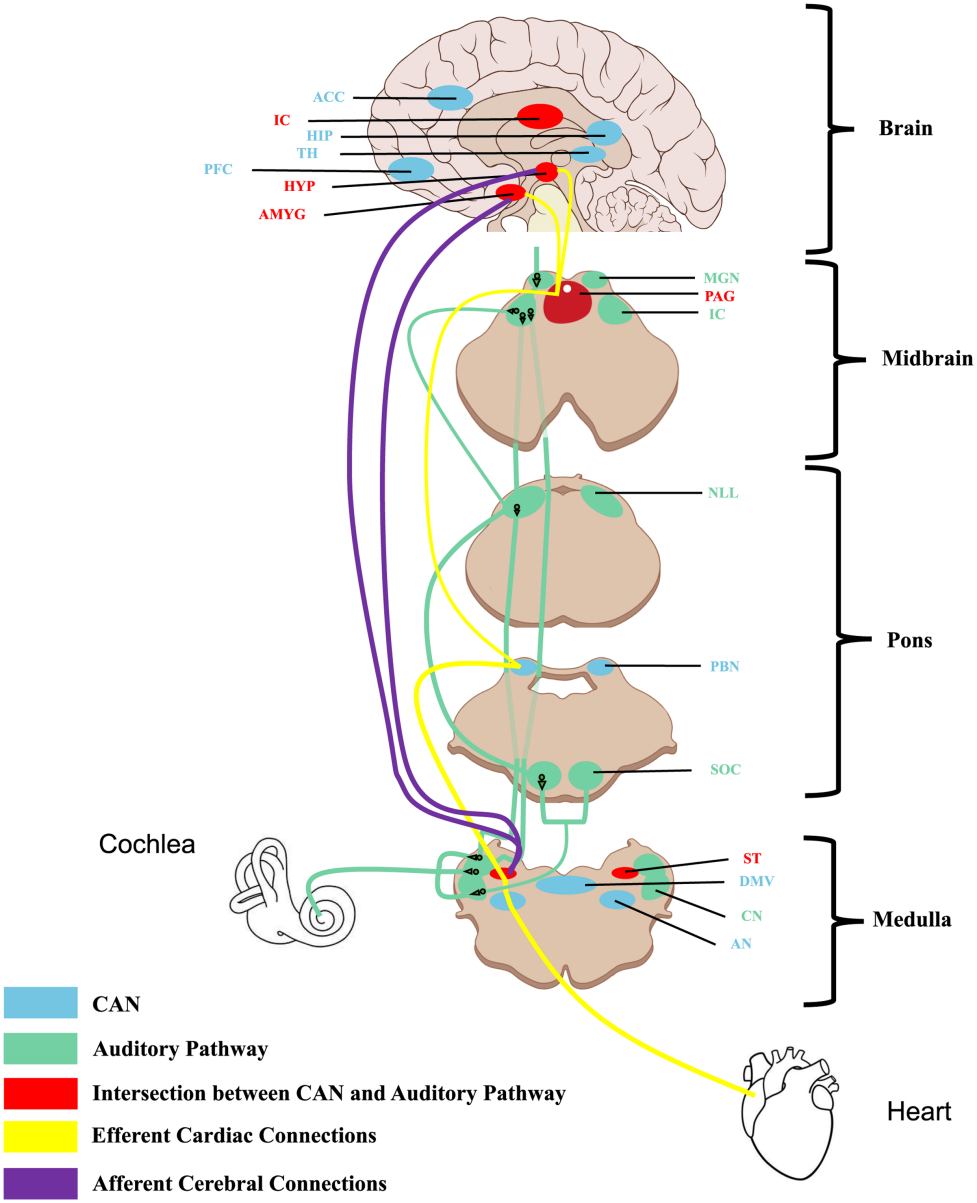
Simplified schematic of the auditory processing pathways and the Central Autonomic Network (CAN) and their interactions. The CAN governs communication between the brain and the heart. It integrates sensory, emotional, and cognitive information to bidirectionally modulate the autonomic nervous system and maintain internal homeostasis, enabling adaptive responses to environmental stimuli. The figure illustrates the main structures of the auditory processing pathway (green) and the CAN pathways, afferent (purple) and efferent (yellow) cardiac, as well as the structures where the two pathways intersect (red).

These transformations are embedded in the Central Autonomic Network (CAN)—a distributed circuit spanning amygdala, anterior insula, cingulate cortex, hypothalamus, and prefrontal areas—that modulates the oscillatory output of brain-stem cardiorespiratory nuclei (Pribram and Melges, 1969; Porges, 2007; Thayer et al., 2009; Beissner et al., 2013). The CAN is anatomically and functionally intertwined with ascending and descending auditory pathways (Figure 2) that project from the cochlea through the inferior colliculus and medial geniculate body to primary and associative auditory cortices (Malmierca, 2006; Leong et al., 2023). Convergence zones in the anterior insula and cingulate cortex interface these auditory circuits with autonomic regulators (Ferraro et al., 2022), while the inferior colliculus integrates auditory and premotor signals, providing a substrate for sensorimotor entrainment (Malmierca, 2006). The spatial proximity and dense interconnectivity of CAN and auditory pathways thus offer a plausible route by which music can modulate heart-rate dynamics and, by extension, emotional state (Marcomini et al., 2018).

Recent tract-tracing and functional-connectivity data further underscore the overlap between auditory, autonomic, and limbic-language networks (Chen et al., 2008; Wu et al., 2011; Garrett et al., 2023), highlighting the intrinsically integrative, visceral nature of brain function (Regaçone et al., 2014). This convergence supports a unified account in which musical experience influences, and is reciprocally shaped by emotion, cognition, and autonomic control. Koelsch and Jäncke (2015) provide a comprehensive review of the effects of music on cardiovascular functioning. Among the findings discussed are reductions in heart rate variability (HRV), evident in both time- and frequency-domain measures, and increases in heart and respiratory rates during exposure to emotionally arousing relative to relaxing music. The review further highlights the role of music in eliciting both positive and negative emotions and examines its effects in the context of mental health conditions such as anxiety and depression.

### Music and interpersonal cardiac synchronization

Early reports identified HR as the physiological signal most tightly coupled to musical rhythm, possibly because cardiorespiratory rhythms can entrain neuronal oscillations operating within overlapping frequency bands (Dainow, 1977). Subsequent work confirmed a robust tempo-dependent effect on HR: brisk music accelerates HR, whereas slower music decelerates it (Agrawal et al., 2013; Mok and Wong, 2003), although phase coupling or synchronization with musical beats has not been demonstrated. This phenomenon underpins many music-therapy protocols, which use slow or gradually decelerating pieces to induce relaxation, lower anxiety, and align both electroencephalographic (EEG) rhythms and HR with the musical tempo (Tso et al., 2022). Emotional engagement modulates the strength of this coupling, highly arousing or personally meaningful passages yield larger cardio-autonomic adjustments than neutral material (Sebastiani et al., 2022).

Cardiac synchronization also emerges in group settings. Passive listening to devotional chants increases the coherence of listeners’ cardiorespiratory rhythms (Bernardi et al., 2017), and the magnitude of cardiac inter-subject synchrony (IS-HR) grows with piece duration (Mollakazemi et al., 2019) and with the shared emotional intensity of live performances (Chew et al., 2019; Tschacher et al., 2023). Similar effects appear during active music-making: HR synchrony among singers predicts tighter intonation and phrasing (Müller and Lindenberger, 2011) and is further enhanced when performers maintain light physical contact (Lange et al., 2022). Group improvisation (Gibbs et al., 2023) and collective rhythm execution (Tomashin et al., 2022) likewise benefit from higher cardiac concordance.

EEG–ECG studies show that the degree of IS-HR tracks inter-subject correlation (ISC) of cortical responses: attentive, emotionally engaged listeners exhibit both stronger neural ISC and tighter HR alignment (Schmälzle et al., 2015; Ki et al., 2016). Factors that amplify empathy, excitement, or direct social interaction further elevate both neural and cardiac synchrony (Konvalinka et al., 2011; Goldstein et al., 2017; Palumbo et al., 2017; Stuldreher et al., 2020). Remarkably, HR synchrony can arise even when individuals listen to the same recording in isolation, indicating that a common exogenous rhythm is sufficient to coordinate autonomic fluctuations (Golland et al., 2014).

Mechanistically, HR dynamics participate in a bidirectional brain–heart loop. Motor imagery, for example, can accelerate HR almost as strongly as overt exercise (Wuyam et al., 1995; Koppula et al., 2022), whereas meditative or gratitude-focused cognition slows it (Kyeong et al., 2017). Cross-frequency-coupling models propose that the phase of slow cardiac oscillations (≈1 Hz at rest) can gate the amplitude of faster cortical rhythms, thereby facilitating large-scale neuronal coordination (Klimesch, 2018). Empirical evidence supports both heart-to-brain and brain-to-heart directions of influence. Amplitude–frequency analyses reveal that high-frequency components of HR variability (0.5–4 Hz) can drive EEG power across the canonical delta-to-gamma bands (Sargent et al., 2024). Conversely, state-dependent shifts in cortical activity (e.g., sleep stages, controlled-breathing paradigms) often dominate the causal flow from brain to heart (Lin et al., 2016; Pardo-Rodriguez et al., 2021; Abdalbari et al., 2022). The consensus is therefore that brain–heart interactions are reciprocal and dynamically reweighted across behavioral contexts.

Thus, music can both sculpt cardiac rhythms and be differentially processed depending on the current cardiac–autonomic state. In group settings, such bidirectional coupling may amplify shared physiological states, reinforcing collective attention, emotion, and coordinated action.

### Groove, embodied cognition, and collective physiology

*Groove* is typically defined as the quality of a musical passage that elicits an urge to move in time with the beat (Stupacher et al., 2013). Embodied-cognition theory frames this urge as a consequence of the body’s integral role in perceiving and giving meaning to the world: sensorimotor systems, interoceptive signals, and prior bodily experience collectively shape conceptual knowledge (Fincher-Kiefer, 2019; Leman and Maes, 2014). Within music research this perspective has matured into the field of *musical embodied cognition* (Davis et al., 2012; Perlovsky, 2015).

Motor involvement is therefore bidirectional: music drives the motor system, and covert (or overt) motor simulation colours musical perception. Functional-neuroimaging studies show enhanced activity in the cerebellum, the supplementary and premotor cortices, and the basal ganglia when listeners perceive a beat, even when they remain physically still (Bengtsson et al., 2009; Chen et al., 2008; Grahn and Rowe, 2009; Zatorre et al., 2007). Recent neurophysiological work suggests that the subjective “feel” of groove arises first from bottom-up neural entrainment to rhythmic regularities (Etani et al., 2024) and is then refined by top-down predictive models of meter and phrase structure (Müller et al., 2021). Reciprocal communication between auditory and motor areas allows the brain to (i) internally simulate movement, (ii) align that simulation to the external beat, and (iii) update predictive models when expectations are met or violated — processes that are experienced as pleasure and reward.

At the group level, shared groove may stabilize autonomic activity and encourage prosocial behavior. Historical accounts, from marching armies to communal dances, illustrate how synchronized rhythmic action fosters a sense of unity and collective purpose.

### Interpersonal cardiac synchrony and prosocial behavior

Heartbeat dynamics provide a tractable window on such collective states. Moment-to-moment changes in HR correlate with empathic engagement during social interaction (Steiger et al., 2019). Laboratory studies show that IS of behavior promotes affiliation (Hove and Risen, 2009; Valdesolo and DeSteno, 2011; Launay et al., 2013), and HR-IS tracks these effects: observers’ heartbeats align with those of loved ones in peril (Konvalinka et al., 2011), presumably because coherent cardiac signals facilitate emotion regulation via heart-to-brain afferents (McCraty and Tomasino, 2006).

Physiological concordance is particularly pronounced in music-making. In choirs, oscillatory coupling of cardiac and respiratory rhythms predicts tighter ensemble timing (Müller and Lindenberger, 2011), and light interpersonal touch further amplifies this coupling in empathetic contexts (Goldstein et al., 2017; Lange et al., 2022). Improvising musicians (Gibbs et al., 2023) and participants executing group rhythms (Tomashin et al., 2022) likewise perform better when their HRs converge.

Cardiac coherence during joint activities also forecasts mutual trust and other prosocial outcomes. Higher HR-IS predicts cooperative choices in economic games (Mønster et al., 2016), enhances rapport in psychotherapy dyads (Kodama et al., 2018), and longitudinally predicts adolescents’ prosocial tendencies (Sijtsema et al., 2013). Meta-analytic evidence links IS to a common neural mechanism for social grouping and reward (Rennung and Göritz, 2016; Shamay-Tsoory et al., 2019), and HR-IS has been proposed as a physiological marker of group cohesion (Gordon et al., 2020a; Gordon et al., 2020b). However, interpretation of these findings should be tempered by the methodological heterogeneity that characterizes the interpersonal synchrony field (Gordon and Bartsch, 2026). Cardiac synchrony can be quantified using diverse approaches—including cross-correlation, coherence analyses, phase-based metrics, recurrence methods, and information-theoretic measures—which capture distinct aspects of coupling and may yield partially divergent results (Palumbo et al., 2017; Gordon and Bartsch, 2026). Moreover, in musical settings, it remains challenging to disentangle genuine interpersonal coupling from synchronization driven by a shared external stimulus (Golland et al., 2014). Greater methodological standardization will therefore be important for strengthening cross-study comparisons and cumulative theory building.

Crucially, synchrony often emerges spontaneously: people align movements and physiology even when instructed not to (van Ulzen et al., 2008), and those with stronger prosocial dispositions synchronise more readily than ego-focused individuals (Lumsden et al., 2014). This spontaneous emergence is theoretically important because it may represent a signature of self-organization within human social systems (Arenas et al., 2008; Orsucci and Tschacher, 2025). From a complex-systems perspective, synchronization reflects the emergence of coordinated collective dynamics from local interactions and reciprocal feedback rather than from centralized control (Arenas et al., 2008; Palmer and Demos, 2022). In musical contexts, shared rhythms, mutual adaptation, and continuous sensorimotor exchange may allow independently regulated physiological processes to become temporarily integrated into a higher-order group state. Because rhythmic music is a powerful driver of unintentional synchrony, it likely serves as a cultural tool for nurturing cooperation and shared emotional experience (Clayton et al., 2005; Brown and Jordania, 2013). In turn, HR-IS appears tightly coupled to behavioral coordination (Goldstein et al., 2017) and joint creative effort (Fusaroli et al., 2016), underscoring the bidirectional link between physiological alignment and collective action.

## Conclusion

Music is uniquely positioned to transform individual physiology into collective physiology and thereby catalyze prosocial behavior. Across studies, auditory rhythms entrain heart rate, respiration, electrodermal tone, and cortical oscillations; when listeners share the same musical stimulus, or co-produce it in song, drumming, or dance, those entrained signals become synchronized across people. Heart-rate interpersonal synchrony (HR-IS) is especially robust: it tracks attention and emotional arousal, predicts tighter ensemble performance, and prospectively forecasts trust, empathy, and cooperative choice. Mechanistically, HR-IS sits at the nexus of a bidirectional brain-heart loop in which the Central Autonomic Network integrates auditory–motor predictions with visceral feedback, aligning internal states to an external beat and, in turn, amplifying neural alignment between individuals.

In summary, the evidence supports three claims:

1. *Causal leverage of rhythm*: tempo, metrical clarity, and groove all modulate the strength of physiological coupling, implying that musical structure can be tuned to elicit desired social outcomes (e.g., relaxation vs. mobilization, bonding vs. differentiation).
2. *Autonomic gateway to prosociality*: convergent autonomic rhythms may serve as a low-latency mechanism through which shared affect fosters perspective-taking, cooperative decision-making, and group cohesion.
3. *Bidirectional dynamics*: because heart-to-brain and brain-to-heart influences are reciprocal and state-dependent, musical interventions may either stabilize an existing collective state or shift it toward new affective trajectories.

Future work should move beyond pairwise designs to larger, ecologically valid groups; integrate multimodal imaging (e.g., fNIRS-ECG-motion capture) for causal modelling; and examine how individual differences (age, musical expertise, autonomic flexibility) gate the transition from physiological coupling to durable social change. Particular attention should also be given to the dynamical processes through which synchrony emerges, stabilises, and dissolves over time. Understanding these transitions may help determine whether interpersonal heart synchrony constitutes an emerging marker of self-organization in musical contexts, linking individual physiological regulation to collective social dynamics. Such efforts will clarify how music can be harnessed as a scalable tool for fostering empathy, cooperation, and public health, and which individuals are most likely to benefit from it.
